# Increases in plasma neurofilament light chain and optic nerve sheath diameter are associated with altitude but show little association with each other

**DOI:** 10.1113/EP094003

**Published:** 2026-11-14

**Authors:** Benedikt Treml, Klaus Berek, Sasa Rajsic, Elisabeth Skalla, Selina Sartori, Harald Hegen, Florian Deisenhammer, Zoran Bukumirić, Martin Burtscher, Axel Kleinsasser

**Affiliations:** ^1^ Department of Anaesthesiology Medical University of Innsbruck Innsbruck Austria; ^2^ Department of Neurology Medical University of Innsbruck Innsbruck Austria; ^3^ Department of Radiology Medical University of Innsbruck Innsbruck Austria; ^4^ Department of Anaesthesia and Critical Care Medicine Medical University of Graz Graz Austria; ^5^ Institute of Medical Statistics and Informatics, Faculty of Medicine University of Belgrade Belgrade Serbia; ^6^ Department of Sports Medicine University of Innsbruck Innsbruck Austria

**Keywords:** acclimatization, neurofilament light chain, optic nerve sheath diameter

## Abstract

Plasma neurofilament light chain (NfL) has been reported to increase following acute and repeated exposure to altitude. Independently, optic nerve sheath diameter (ONSD) has been shown to increase at altitude. In this study, we measured NfL and ONSD in parallel before, during and after a 44‐h sojourn at 3100 m, and examined whether these changes were associated. Twelve healthy adults of either sex were transported to 3100 m. Blood samples and sonographic assessment of the ONSD were obtained at baseline and after 24 and 44 h at altitude. Plasma analyses included NfL and glial fibrillary acidic protein (GFAP). The occurrence of acute mountain sickness (AMS) was assessed using the Lake Louise Score. Over 44 h, the age‐ and body mass index‐standardized *z*‐score of NfL increased from 0.88 to 1.17 (median, *P* = 0.050). Concurrently, ONSD increased from 4.30 mm to 4.80 mm (median, *P* = 0.016). Linear regression analysis showed that *z*‐score of NfL, as the dependent variable, was not associated with ONSD, the independent variable (*R^2^* = 0.026, *P* = 0.354). Mixed model linear regression revealed an association but with a small effect size. GFAP, as a marker of blood–brain barrier injury and inflammatory parameters, remained unaffected by exposure to altitude. No participant developed AMS. An altitude of 3100 m is attainable by many individuals, but is associated with reduced oxygen availability and a risk of AMS. At this altitude in our experiment, plasma NfL and ONSD increased *in parallel* but with little to no association over 44 h.

## Abstract (highlights)

- **What is the central question of this study?**
  Hypobaric hypoxia may increase intracranial pressure, and studies have shown that both hypo‐ and normobaric hypoxia can increase blood neurofilament light chain levels and optic nerve sheath diameter: do these changes occur together in the same individuals?
- **What is the main finding and its importance?**
  Neurofilament light chain concentrations and optic nerve sheath diameter both increased individually, but the changes were hardly associated. The optimal timing of measurements remains unclear, as neurofilament light chain changes may be delayed. Altitude exposure caused measurable cerebral changes that appeared to be well compensated.

## 1 INTRODUCTION

When exposed to high altitude, the reduced partial pressure of oxygen in the atmosphere leads to a decrease in arterial oxygen partial pressure. Despite compensatory hyperventilation, individuals may develop acute mountain sickness (AMS), a usually benign symptom complex including headache, nausea and fatigue (Gatterer et al., 2024). AMS is usually self‐limiting (Gatterer et al., 2024).

### 1.1 Intracranial pressure at altitude

High‐altitude cerebral oedema (HACE) is a potentially fatal condition that may represent either a progression of AMS or a distinct clinical entity (Turner et al., 2021). In HACE, intracranial pressure (ICP) is elevated (Turner et al., 2021). In plain AMS, however, ICP elevation has not been considered the primary pathophysiological mechanism based on studies using magnetic resonance imaging (Bartsch et al., 2004) or distortion product otoacoustic emissions (Olzowy et al., 2014). Nevertheless, an increase in ICP in AMS appears plausible (Wilson et al., 2014) although the mechanisms and extent of ICP changes in individuals remain unclear. In this experiment, we focused on two strictly independent markers of ICP: neurofilament light chain (NfL) and optic nerve sheath diameter (ONSD).

### 1.2 NfL as a biomarker of an increased ICP

NfL is an extremely sensitive biomarker of neuronal damage; however, NfL does not provide specificity regarding the underlying cause or the anatomical location of the pathology: NfL is part of the cytoskeleton in axons (La Civita et al., 2025). When axons are damaged by disease or trauma, NfL leaks into the cerebrospinal fluid and to a lesser extent into the blood (La Civita et al., 2025). Rather than being specific and therefore indicative for a certain condition, NfL is currently used to gauge activity, progress or prognosis of a known pathologic process.

Interestingly, in the setting of isobaric hypoxia (a surrogate for high altitude), NfL increases and was shown to be associated with AMS (Berek et al., 2024). However, isobaric (‘chamber’) hypoxia and real, hypobaric hypoxia are not fully comparable in this respect: In a first field study, Sareban and coworkers (2021) found that plasma NfL levels were increased after active ascent to 4559 m, but a correlation to AMS could not be found.

Also chronic, repeated exposure to altitude may be the cause of elevations in NfL without apparent disease or clinical symptoms: in Canadian military pilots and aircrew who were repeatedly exposed to high altitude, NfL was elevated in comparison to those who remained on the ground (Rhind et al., 2024). Accordingly, there is an association of NfL leak with iso‐ and hypobaric hypoxia.

### 1.3 Optic nerve sheath diameter as an indicator of an increased ICP

ONSD is the transverse width of the dural sheet surrounding the optic nerve at 3 mm behind the globe (Malky et al., 2025; Montorfano et al., 2021). ONSD can be measured using ocular ultrasound and used as a non‐invasive surrogate marker of ICP (Malky et al., 2025; Montorfano et al., 2021). ONSD was found to increase at altitude in a number of field studies (Kanaan et al., 2015; Keyes et al., 2013; Lawley et al., 2012; Willmann et al., 2014), but these field studies failed to give consistent evidence that ONSD reflects an increased ICP in AMS (Kanaan et al., 2015; Keyes et al., 2013; Lawley et al., 2012; Willmann et al., 2014). These varying results may be due to inconsistent angles of ultrasound transducer (also the examiner is hypoxic), due to a poor standardization and due to the poorer image quality which contemporary transportable devices deliver. To date, ONSD field studies have produced inconsistent results.

In contrast, ONSD measurements in lowland settings yield consistent results: in ultrasound studies performed in healthcare facilities, an ONSD of 5.82 mm indicates an ICP greater than 20 mmHg as shown in a meta‐analysis by Montorfano and coworkers (2021). Only slightly deviating, in a recent work by Malky and coworkers (2025), an ONSD of 5.7 mm was shown to be a robust predictor of an elevated ICP in a condition called idiopathic intracranial hypertension.

### 1.4 Uncertain relationship between NfL and ONSD

A potential association between NfL and ONSD as markers of ICP at high altitude has not yet been elucidated. Given that NfL is elevated in idiopathic intracranial hypertension (Malky et al., 2025; Montorfano et al., 2021), in normobaric hypoxia (Berek et al., 2024), and following active ascent to 4559 m (Sareban et al. 2021), and that ONSD serves as a non‐invasive surrogate measure of ICP, it is possible that elevations in NfL and ONSD are associated at high altitude.

Hence, we hypothesized a positive statistical association between NfL and ONSD during high‐altitude exposure, which constituted our primary research question.

Secondary endpoints included altitude‐related changes in glial fibrillary acidic protein (GFAP), reflecting astrocyte and blood–brain barrier injury, as well as concurrently assessing whether background inflammatory processes could account for expected increases in NfL, GFAP and ONSD.

## 2 METHODS

This experiment was performed as part of a study of early acclimatization to altitude which was published recently (Skalla et al., 2026), where also a more detailed description of the methods is provided. In brief, 12 healthy subjects were passively brought to the Sonnblick Observatory, Austria (www.sonnblick.net/en/) at 3100 m above sea level. All assessments or measurements were performed or taken before and after 24 and 44 h of exposure to altitude.

### 2.1 Barometric pressure

At the time of the experimental runs, concurrent barometric pressure data were obtained from the official website of the Institute of Meteorology, Vienna, Austria (ZAMG, zamg.ac.at), a service that is no longer active in its original form.

### 2.2 Participant recruitment

Participants were recruited on a voluntary basis and provided informed consent for repeated blood sampling via individual punctures of the radial artery and either a forearm or cubital vein. We aimed to enrol healthy, physically active subjects; the main inclusion criterion was age ≥18 years. Key exclusion criteria included pre‐existing medical conditions, particularly neurological disease, poor altitude tolerance or a history of altitude‐related illness. The experiment was conducted in April 2023.

Informed written consent was obtained from all 12 participants in this study. The study protocol was approved by the Ethics Committee of Innsbruck Medical University (reference number: 1111/2022). This work conforms with the standards set by the *Declaration of Helsinki* (last modified in 2013).

### 2.3 Subjects

The examined 12 healthy volunteers were aged from their 30s to their 70s, with five females and seven males. Mean age was 50 years and mean body mass index was 23.0. All lived in lowland areas at about 600 m above sea level, all were non‐smokers and none had a recent (within the previous month) exposure to high altitude. In particular, no one had a history of a neurological disease.

### 2.4 Measurements, general

We took all baseline measurements at an altitude of 575 m (University Hospital of Innsbruck, Innsbruck, Austria), barometric pressure on the day measurements were taken was 715 Torr. Participants were then transported by car to Kolm Saigurn, Salzburg, Austria and further on by using the funicular to the Sonnblick Observatory, Austria at 3100 m above sea level (www.sonnblick.net/en/). Barometric pressure at the observatory was 511 Torr upon arrival and varied only slightly over the study period. Measurements and blood samples at the observatory were taken after continuous exposure to altitude for 24 and 44 h.

### 2.5 Arterial blood gas analyses and calculation of oxygen delivery

Arterial blood samples were taken and immediately analysed using a blood gas analyser (Radiometer Copenhagen, ABL80 FLEX, Radiometer MP, Vienna, Austria). Parameters observed in this experiment included arterial partial pressure of oxygen ($P_{aO_{2}}$) and the fraction of the oxygenated haemoglobin (${F_{O_{2}}}_{\mathrm{Hb}}$). These variables were used to calculate oxygen delivery ($D_{O_{2}}$). $D_{O_{2}}$ was calculated using the following equation:

$$D_{O_{2}}=\mathrm{Cardiacindex}\cdot \left[10\cdot \left(\mathrm{Hb}\cdot \;F_{O_{2}\mathrm{Hb}}\cdot 1.34\right)+\left(P_{aO_{2}}\cdot 0.003\right)\right]$$

and is given in ml min^−1^ m^−2^.

### 2.6 Circulatory measurements

Circulatory measurements included cardiac output and index using a non‐invasive monitor (CNAP, CN Systems, Graz, Austria) which delivers reliable measurements (Dewhirst et al., 2013).

### 2.7 Altitude sickness

Questionnaires for altitude‐associated sickness were answered at 575 m and after 24 and 44 h at 3100 m. In this trial, we used the 2018 edition of the Lake Louise Score (Roach et al., 2018).

### 2.8 Assessment of ONSD

In our study, ONSD was measured 3 mm posterior to the globe in a sagittal plane (Malky et al., 2025; Montorfano et al., 2021) and three measurements performed on the same eye were averaged. Sonography for OSND was performed by a board‐certified radiologist. A Canon Viamo SV7 (Canon, Vienna, Austria) equipped with a high‐frequency linear array probe was used.

### 2.9 Analyses of NfL and GFAP

Raw plasma NfL (pg ml^−1^) was assessed using a Lumipulse G 1200 (Fujirebo Diagnostics, Vienna, Austria) (La Civita et al., 2025). Plasma NfL *z*‐score was then calculated using

$$\mathrm{NfL}\;z\;\mathrm{score}=\frac{\mathrm{Measured}\;\mathrm{NfL}-\mathrm{Predicted}\;\mathrm{NfL}\left(\mathrm{age},\;\mathrm{BMI}\right)}{\mathrm{Residual}\;\mathrm{SD}}$$

based on a previously published control cohort correcting absolute NfL concentrations for age sex and BMI (Benkert et al., 2022).

Using NfL *z*‐scores was of particular importance since the age of our volunteers ranged from mid 30s to mid 70s.

Other parameters assessed in the blood plasma included GFAP (Ogłodek & Bar, 2026), interleukins 6 and 10, C‐reactive protein and erythropoietin.

### 2.10 Statistical analyses and software used

We examined the effects of 24‐ and 44‐h altitude exposure on ONSD and a series of blood plasma variables. For this, we applied Friedman's two‐way analysis of variance, as the parameters were repeatedly measured under different conditions and times within the same subjects, and the data were not normally distributed. A Bonferroni correction was used to adjust for type 1 error. A *P*‐value ≤0.05 was considered significant.

The association between ONSD and NfL *z*‐score was assessed using linear regression, incorporating all available data for both variables. ONSD was treated as the independent variable (*x*‐axis) and *z*‐score NfL as the dependent variable (*y*‐axis). In addition, Spearman's rank correlation coefficient was used to assess the association between changes in ONSD and NfL values at 24 and 44 h relative to baseline. Changes in NfL *z*‐score and ONSD were analysed using a linear mixed‐effects model fitted by REML. Time (baseline, 24 and 44 h) was included as an ordered continuous fixed effect, with Participant as a random intercept. The model assumed normally distributed random intercepts and independent residuals with constant variance, corresponding to compound symmetry within participants. Missing values were not imputed, and all available measurements were analysed under the missing‐at‐random assumption. The treatment of time is described by stating that measurement occasion was included as a numeric fixed effect coded 1, 2 and 3, thereby assuming a linear trend across the three ordered measurement occasions. The covariance assumptions are described by the participant‐specific normally distributed random intercepts and conditionally independent residuals with constant variance, which induce a compound‐symmetry covariance structure for repeated measurements within participants.

Statistical analyses were performed by using SPSS (Version 22.0 released 2013; IBM Corp., Armonk, NY, USA). Data are given as median (minimum–maximum) or mean when appropriate (ONSD threshold, biometrics). Plotting was done using GraphPad Prism 8 (GraphPad Software, San Diego, CA, USA) and SPSS.

## 3 RESULTS

Results are displayed in Figures 1, 2, 3 and in Table 1. All subjects tolerated the experiment well. Three blood samples were lost due to technical problems (thawing), such that with interleukin (IL)‐6 and IL‐10, C‐reactive protein, erythropoietin and NfL and GFAP, *n* at the lowland measurement was 10; after 24 h at altitude *n* was 12 and after 44 h *n* was 11. Two more erythropoietin essays could not be performed after 44 h since the sample volume was small and NfL and GFAP essays were preferred. The *n* for erythropoietin was thus 10 at the lowland time point, 12 at the 24 h time point and 9 at the 44 h time point.

**FIGURE 1:**
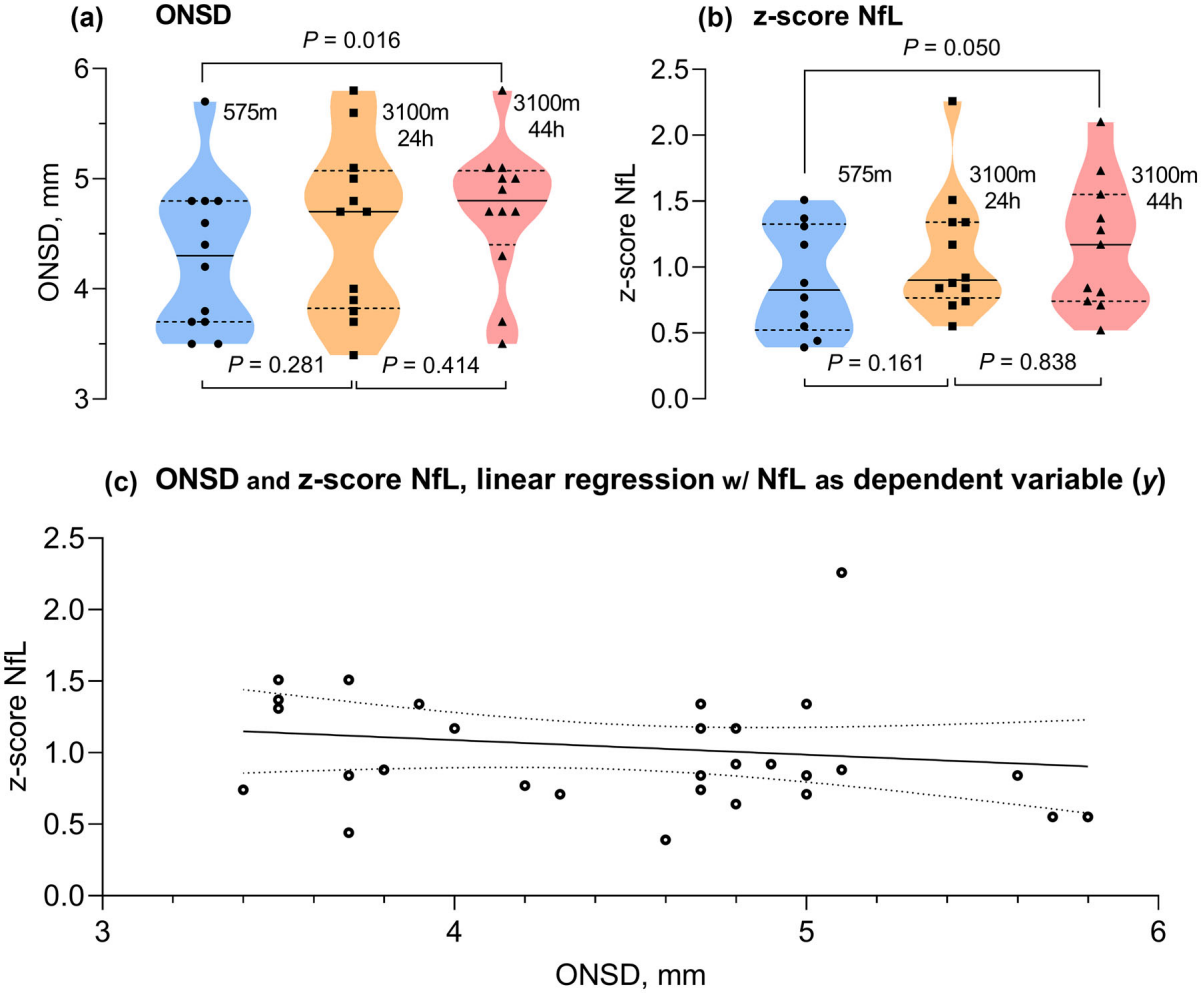
Optic nerve sheath diameter and *z*‐score neurofilament light at altitude. (a, b) Optic nerve sheath diameter (ONSD; a) and *z*‐score neurofilament light (*z*‐score NfL; b) at baseline (lowland measurement, *n* = 10) and at 24 h (*n* = 12) and 44 h (*n* = 11) after exposure to 3100 m. Violin plots were used to provide a smoothed estimate of the data distribution. The continuous line denotes the median while quartiles (25 and 75) are displayed using dashed lines. (c) Linear regression of *z*‐score NfL (dependent variable) on ONSD; included are all data points to show the distribution (33 points, some are not seen due to overlapping). The dotted curves enclose the 95% confidence band.

**FIGURE 2:**
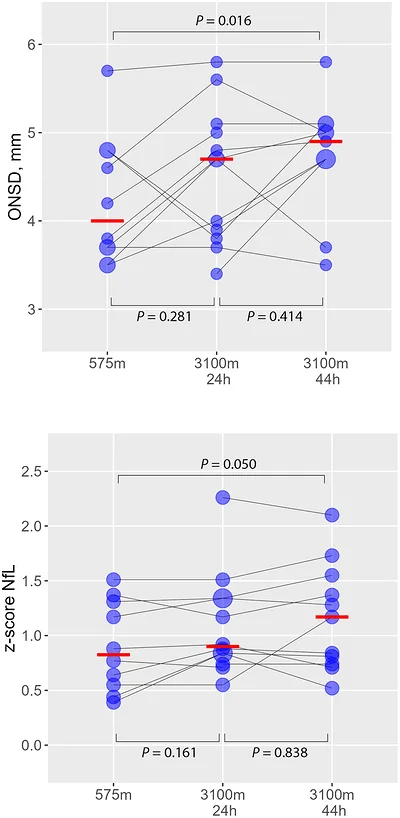
Individual trajectories of Figure 1a,b. (a) ONSD (*n* = 12/12/12) and (b) *z*‐score NfL (*n* = 10/12/11). SPSS‐generated graph; red bars indicate median.

**FIGURE 3:**
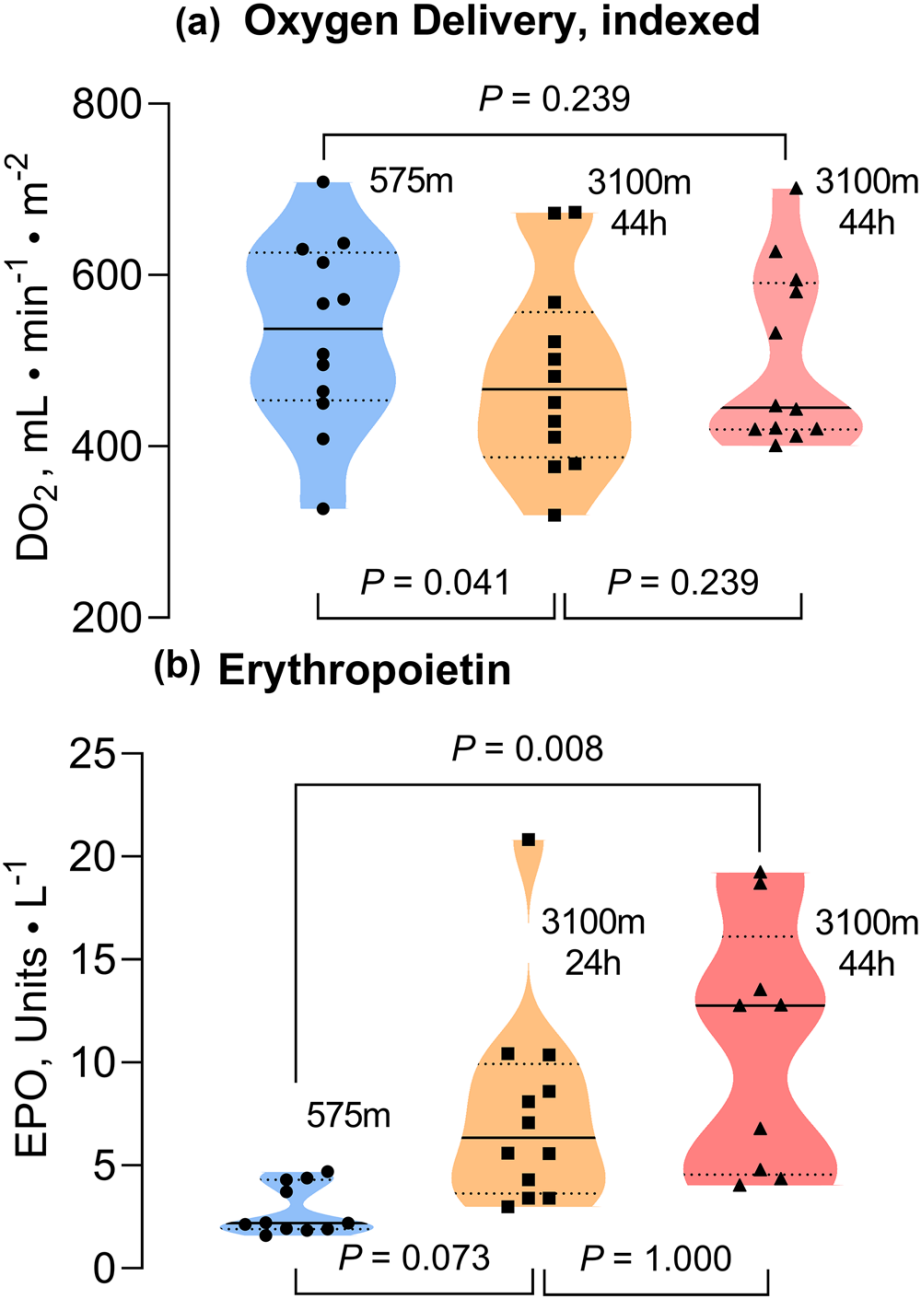
Hypobaric hypoxia. Influence on oxygen delivery (a) and erythropoietin (b). Oxygen delivery index ($D_{O_{2}}$) (*n* = 12/12/12) (a) and erythropoietin (EPO) (b) at baseline (lowland measurement, *n* = 10) and at 24 h (*n* = 12) and 44 h (*n* = 9) after exposure to 3100 m as violin plots. The continuous line denotes the median while quartiles (25 and 75) are displayed using dotted lines.

**TABLE 1:** Oxygenation, neurofilament light, GFAP and inflammatory markers before and after passive ascent to 3100 m.

Variable	575 m	After 24 h at 3100 m	After 44 h at 3100 m
${F_{O_{2}}}_{\mathrm{Hb}}$ (%)	95.5 (94.1–95.9)	90.1 (88.9–90.7)	90.5 (89.0–91.5)
NfL raw (pg ml^−1^)	11.3 (9.7–14.2)	12.7 (10.5–15.3)	13.7 (11.3–15.2)
GFAP (pg ml^−1^)	24.9 (19.6–27.0)	22.1 (18.7–31.6)	22.1 (16.4–26.8)
IL‐6 (pg ml^−1^)	1.0 (0.6–1.0)	1.0 (0.1–1.0)	1.0 (0.0–1.0)
IL‐10 (pg ml^−1^)	0.1 (0.1–0.2)	0.1 (0.1–0.1)	0.3 (0.1–0.3)
CRP (mg L^−1^)	0.02 (0.01–0.02)	0.01 (0.01–0.04)	0.02 (0.02–0.03)

*Note*: Parameters are given as median (25–75 quartile). With NfL, GFAP, IL‐6 and IL‐10, *n* = 10 at the 575 m measure point, *n* = 12 at the 24 h measure point and *n* = 11 at the 44 h measure point.

Abbreviations: CRP, C‐reactive protein; ${F_{O_{2}}}_{\mathrm{Hb}}$, fraction of oxygenated haemoglobin; GFAP, glial fibrillary acidic protein; IL‐6 and IL‐10, interleukin 6 and 10.

### 3.1 AMS

AMS was not detected using 2018 Lake Louise Score (Roach et al., 2018).

### 3.2 NfL

Plasma raw NfL is shown in Table 1. Plasma *z*‐score NfL is shown in Figure 1b,c. Differences between lowland NfL *z*‐core and 44 h at altitude were significant. Levels of significance are illustrated in Figure 1.

### 3.3 ONSD

ONSD measurements are shown in Figure 1a,c. Sonography of the ONSD revealed one volunteer with an ONSD of above 5 mm before ascent, after 24 h at altitude four volunteers already had an ONSD above 5 mm and after 44 h at altitude 5 out of 12 volunteers had an ONSD greater than 5 mm. Only 1 out of 12 participants reached an ONSD of 5.82 mm, which corresponds to an intracranial pressure of 20 mmHg according to Montorfano et al. (2021), during the sojourn to 3100 m in this study. Nine out of 12 volunteers showed an increase in ONSD. Levels of significance are presented in Figure 1.

### 3.4 Correlation of pre to post changes

Analysed as paired repeated measurements of changes in values at 44 h relative to baseline, the greater the change in ONSD, the smaller the change in NfL [*r*_s_ = −0.83 (95% CI −0.96 to −0.38); *P* = 0.005]. There was no association between changes in ONSD and NfL at 24 h relative to baseline [r_s_ = 0.17 (95% CI −0.52 to 0.72); *P* = 0.647].

The question arises, do participants with larger increases in ONSD also show larger increases in NfL from baseline to 24 and 44 h? The answer is no. At 24 h, participants with larger increases in ONSD did not show correspondingly larger increases in NfL, as the correlation between the change‐from‐baseline scores was weak and not statistically significant (Spearman's rank correlation coefficient *r*_S_ = 0.17, 95% CI −0.52 to 0.72; *P* = 0.647). At 44 h, a strong inverse correlation was observed (*r*_S_ = −0.83, 95% CI −0.96 to −0.38; *P* = 0.005), indicating that participants with larger increases in ONSD tended to have smaller increases, or greater decreases, in NfL. Given the relatively small sample size, the inverse association at 44 h should be interpreted cautiously as an exploratory finding. This also applies to the effects sizes: given the small sample, isolated *P*‐values are not sufficient to judge the magnitude or physiological relevance of the findings.

### 3.5 Association of *z*‐score NfL with ONSD examined with regression and mixed model analysis

A graphical display of the association of *z*‐score NfL and ONSD is displayed in Figure 1c. The association between all parallel NfL *z*‐score and ONSD assessments was first examined using linear regression. *z*‐Score NfL as the dependent variable was not associated with ONSD resulting in *R^2^* = 0.026, *F* = 0.88, *P* = 0.354 in 34 pairs.

In addition, we fitted a linear mixed‐effects model accounting for repeated measurements. ONSD was positively associated with *z*‐score NfL (*b* = 0.086, *P* = 0.032). Although this relationship reached statistical significance, the effect size was small, indicating only a weak association between ONSD and NfL. Accordingly, *b* = 0.086 (regression coefficient) indicates that for each 1 mm increase in ONSD, *z*‐score NfL increases on average by 0.086 units.

### 3.6 Exposure to hypobaric hypoxia: oxygen delivery and erythropoietin release

Oxygen delivery indexed to body surface area ($D_{O_{2}}$) is shown in Figure 3a. $D_{O_{2}}$ decreased after 24 h of hypoxia [537 to 446 mL min^−^^1^ m^−^^2^ (median)], indicating reduced oxygen availability at the tissue level. In response to this decrease in $D_{O_{2}}$, erythropoietin concentrations increased from a median of 2.18 to 7.82 U L^−^^1^ after 24 h and further to 12.76 U L^−^^1^ after 44 h.

### 3.7 Inflammation

To exclude inflammation as a potential cause of changes in ONSD and *z*‐score NfL, we measured IL‐6, and IL‐10, as well as C‐reactive protein and GFAP; all markers showed no evidence of inflammation in any of the volunteers (Table 1).

## 4 DISCUSSION

In this study investigating parallel changes in NfL and ONSD, we observed increases in both parameters after 44 h of high‐altitude exposure. However, contrary to our initial hypothesis, we found little to no association between these two variables. GFAP and inflammatory parameters remained unchanged.

### 4.1 Why were we hopeful that changes in NfL were linked to changes in ONSD?

Altitude illness is common and increases with elevation, with AMS affecting 17.8% at 2800 m and 38% at 3500 m (Mairer et al., 2009). Headache occurs in ∼31% during the first night at altitude and is associated with migraine and hypoxaemia (Burtscher et al., 2011). Given the uncertain relationship between AMS and ICP, we assessed surrogate ICP markers, including ONSD and plasma NfL.

NfL increases after high‐altitude exposure or isobaric hypoxia (Sareban et al. 2021; Berek et al., 2024; Rhind et al., 2024). ICP is associated with papilloedema and ONSD enlargement, although not specifically at altitude (Knoche et al., 2023). ONSD responses to altitude are inconsistent (Kanaan et al., 2015; Keyes et al., 2013; Lawley et al., 2012; Willmann et al., 2014).

An association between NfL and ONSD would support indirect ICP estimation and central nervous system assessment in field settings. However, our data do not provide robust support for ONSD as a proxy for NfL under hypobaric hypoxia. A mixed‐effects linear regression revealed a statistically significant association, but the effect size was small: a 0.086 change in NfL *z*‐score per 1 mm change in ONSD. The range of NfL *z*‐scores was 0.39 to 2.26.

### 4.2 Interpretation of our findings

Our findings for NfL are consistent with the absence of neuronal injury (Khalil et al., 2018, 2024), as a *z*‐score ≥2 (corresponding to the 97.5th percentile of an age‐adjusted reference population) was exceeded only in one volunteer in his thirties, who exhibited no other symptoms.

We observed an increase in ONSD, which did not reach the mean 5.7 mm threshold corresponding to 20 mm Hg ICP (Montorfano et al., 2021) indicating a mild elevation in ICP.

GFAP levels remained stable, providing no indication of astroglial activation or astrocyte‐driven neuroinflammation (Ogłodek & Bar, 2026).

In this way, the physiological implications of our findings are that hypobaric hypoxia triggers mild neuroaxonal stress without astrocytic damage, wherein the concurrent ONSD dilation serves as a surrogate marker for sub‐clinical white matter oedema and mechanical tissue strain.

Taken together, using three independent variables, there is a measurable effect of 44 h of altitude exposure on the brain, which in turn appears to be well compensated as indicated by the negative Lake Louise score.

### 4.3 Evaluation of changes in NfL levels at altitude

NfL is highly sensitive but largely non‐specific. Contextualizing our results against other studies is therefore valuable. Comparisons with our previous isobaric study (Berek et al., 2024) would not deliver conclusive results since exposure was shorter (12 h in the chamber vs. 24–44 h at real altitude) and hypoxic provocation was milder in the present hypobaric study (3100 versus 4500 m). Time of exposure and the type of hypoxia (isobaric vs. hypobaric) thus appear relevant. Another difference was the assay: in the isobaric study, a single molecule array (SIMOA) technique was used (Berek et al., 2024).

An interesting field study after active ascent to Capanna Margherita (4559 m) (Sareban et al. 2021) is more directly comparable to our previous isobaric study (Berek et al., 2024), as hypoxic challenge and assay methodology were similar. Raw serum NfL levels in the hypobaric hypoxia study were higher, including the lowland time point at 423 m, and also the amplitude of change seemed higher (Sareban et al. 2021) when compared with the initial hypoxic run of the isobaric study. The chamber study showed an association between NfL and AMS, whereas the hypobaric exposure study did not, highlighting the need for controlled studies across altitudes. Also active versus passive ascent may play a role, which is unclear at the moment.

Both our passive ascent study and the work of Sareban et al. (2021), using active ascent to Capanna Margherita, found no association between plasma NfL increases and AMS. In contrast, our recent isobaric study without physical activity demonstrated such an association (Berek et al., 2024). Current evidence for associations between NfL and AMS remains limited.

### 4.4 Relevance of the altitude used

With growing interest in winter sports and the effects of climate change, skiers and recreational athletes increasingly seek higher‐altitude resorts. In the European Alps, 40 winter sports resorts offer alpine skiing from top stations above 3000 m that are accessible by cable car (www.skiresort.info). Hundreds take on ski touring along the Haute Route from Chamonix to Zermatt which exposes mountaineers to altitudes above 3000 m for approximately 1 week. The incidence of AMS in the Alps, as elsewhere, depends on the altitude reached. In resorts where high altitudes are readily accessible, such as Zermatt, AMS incidence rises sharply once altitudes of approximately 3000 m are exceeded. Maggiorini et al. (1990) reported AMS incidences in the Swiss Alps of 9 % at 2850 m, 13 % at 3050 m and 34 % at 3650 m. Similarly, Mairer et al. (2009) reported an altitude‐dependent AMS incidence in the Eastern Alps: 6.9% at 2200 m, 9.1% at 2500 m, 17.4% at 2800 m and 38.0% at 3500 m.

### 4.5 Limitations

In the present study, we investigated a small and heterogeneous cohort at moderate altitude. Increasing the number of participants and studying them at several altitudes might yield more conclusive results. However, altitudes at about 3000–3500 m are of particular relevance to many recreational skiers and snowboarders.

### 4.6 Future research

Our results lead us to new questions. As discussed above, we cannot be sure whether isobaric hypoxia as in our previous study (Berek et al., 2024) is a suitable background model in the research of ICP and associated biomarkers. We suggest that future research might be more conclusive if performed at real altitude in order to separate decompression effects from those produced by hypoxia alone. Also, various altitudes above 2500 m need to be examined. Time points approximately 3 days after the initial hypoxic exposure are of particular interest, as the peak in NfL occurs with a 72‐h delay in a pig model of hypoxia (Kyng et al., 2023). Furthermore, optical coherence tomography of the eye may show structural changes to the retina (like oedema) and optic nerve head swelling secondary to increases in ICP.

### 4.7 Summary

Exposure to 3100 m over 44 h leads to parallel increases in NfL and ONSD, but there was no statistical association detectable between the two. However, as both parameters were affected by altitude exposure, further controlled studies at various altitudes (above and below the 3100 m used in this work) are warranted. This may help provide further insight into how the brain responds to high‐altitude exposure.

## AUTHOR CONTRIBUTIONS

Benedikt Treml and Klaus Berek contributed equally to this work. Martin Burtscher, Klaus Berek, Benedikt Treml, Selina Sartori and Axel Kleinsasser conceptualized the study. Benedikt Treml was responsible for assistance with ethics and organization of the experiment, Elisabeth Skalla organized all aspects of sonographic examinations together with Axel Kleinsasser. Benedikt Treml took care of all data recording and processing together with Selina Sartori and Sasa Rajsic. Sasa Rajsic, Harald Hegen, and Florian Deisenhammer created ways to store, transport and appropriately analyse data at high altitude. Martin Burtscher performed a great part of the lowland examinations together with Klaus Berek. Sasa Rajsic organized workflow at altitude and assured data quality. Axel Kleinsasser and Martin Burtscher took care of data interpretation. Benedikt Treml, Klaus Berek, Axel Kleinsasser, Selina Sartori, Harald Hegen, Florian Deisenhammer, and Martin Burtscher were responsible for manuscript editing. Zoran Bukumirić was responsible for statistics. All listed authors approved the final version of the manuscript; agreed to be accountable for all aspects of the work in ensuring that questions related to the accuracy or integrity of any part of the work are appropriately investigated and resolved; and all persons designated as authors qualify for authorship, and all those who qualify for authorship are listed.

## CONFLICT OF INTEREST

None declared.

## FUNDING INFORMATION

None.

## GENERATIVE AI STATEMENT

No generative AI tools were used in the preparation of this manuscript.

## Data Availability

All data supporting the results presented in the manuscript are included in the manuscript figures.
